# Rosai-Dorfman Disease: A Differential Diagnosis for Tonsillar Asymmetry

**DOI:** 10.7759/cureus.108921

**Published:** 2026-05-15

**Authors:** Irmak Sakin, Dogan Yildiz, Lina Al Omari, Charlotte Harries, Basil Al Omari

**Affiliations:** 1 Otolaryngology-Head and Neck Surgery, James Paget University Hospitals, Great Yarmouth, GBR; 2 Otolaryngology-Head and Neck Surgery, Norfolk and Norwich University Hospitals, Norwich, GBR; 3 Haematology, Norfolk and Norwich University Hospitals, Norwich, GBR; 4 Otolaryngology-Head and Neck Surgery, James Paget University Hospital, Great Yarmouth, GBR; 5 Histopathology, Norfolk and Norwich University Hospitals, Norwich, GBR

**Keywords:** adult tonsils, lymphadenitis, pet-ct scan, rosai-dorfman disease, rosai-dorfman syndrome, sinus histiocytosis, tonsillar asymmetry, tonsillar hypertrophy, tonsillectomy

## Abstract

Rosai-Dorfman disease (RDD) is a rare histiocytic disorder that classically presents with massive cervical lymphadenopathy. We report the case of a 38-year-old Indian man referred to the ear, nose, and throat (ENT) clinic via the urgent cancer pathway with a persistent sore throat and unilateral tonsillar hypertrophy, without cervical lymphadenopathy. Clinical examination demonstrated an isolated, firm, non-ulcerative swelling of the right tonsil. Cross-sectional imaging confirmed tonsillar asymmetry without evidence of local invasion or metastatic disease, although increased uptake was noted on positron emission tomography (PET). The patient underwent bilateral tonsillectomy, and histopathological analysis demonstrated large histiocytic cells with immunohistochemistry positive for S100 and CD68 and negative for CD1a, confirming RDD. Subsequent multidisciplinary follow-up identified additional manifestations of the disease, including lung nodules and cutaneous lesions, and the patient remained under the care of the haematology histiocytosis clinic. This case highlights the importance of considering RDD in the differential diagnosis of tonsillar asymmetry and its implications for optimising multidisciplinary patient care.

## Introduction

We report the case of a 38-year-old man of Indian ethnicity who was referred to the ear, nose, and throat (ENT) clinic via the urgent cancer pathway with a sore throat and unilateral tonsillar enlargement. Following thorough clinical evaluation and appropriate diagnostic investigations, he underwent bilateral tonsillectomy for definitive histological assessment. Histopathological examination confirmed Rosai-Dorfman disease (RDD), a rare non-Langerhans cell histiocytosis.

RDD, also known as sinus histiocytosis with massive lymphadenopathy, is characterised by the overproduction and accumulation of histiocytes that are CD68-positive, S100-positive, and CD1a-negative within affected tissues. Historically considered a benign and often self-limiting inflammatory condition, recent studies have identified recurrent mutations involving the mitogen-activated protein kinase (MAPK) pathway in 30-50% of cases, leading to its recognition by the World Health Organization in 2022 as a histiocytic neoplasm [1].

Due to its rarity, the clinical spectrum and treatment outcomes of RDD remain incompletely defined. It most commonly presents with painless massive cervical lymphadenopathy in young adults, raising suspicion for lymphoma, but may also involve mediastinal, inguinal, and retroperitoneal lymph nodes, as well as extranodal sites such as the skin, nasal cavity, bone, orbit, kidneys, and central nervous system [2]. 

Despite its recent reclassification as a histiocytic neoplasm, RDD generally carries an excellent prognosis and can often be managed with active surveillance alone, particularly in asymptomatic patients. However, relapses may occur, and management frequently requires a multidisciplinary approach because of its heterogeneous clinical manifestations. When treatment is indicated, first-line therapy typically involves corticosteroids. In refractory or relapsing disease, steroid-sparing agents, immunomodulatory therapies, or targeted treatments may be considered. Surgical intervention is primarily reserved for diagnostic biopsy, although it may also be curative in unifocal disease or used for debulking, particularly in cases involving airway compromise [2].

## Case presentation

A 38-year-old male patient was referred to the ENT clinic with a persistent sore throat and asymmetrical tonsillar enlargement. The past medical history included ulcerative colitis (UC), for which he was under gastroenterology follow-up, enteropathic arthritis, chronic right-sided headaches of unclear aetiology under neurological review, left submandibular sialolithiasis planned for surgical removal, previous tongue-tie release, and mild asthma. He had no smoking history and a family history of colitis.

**Figure 1:**
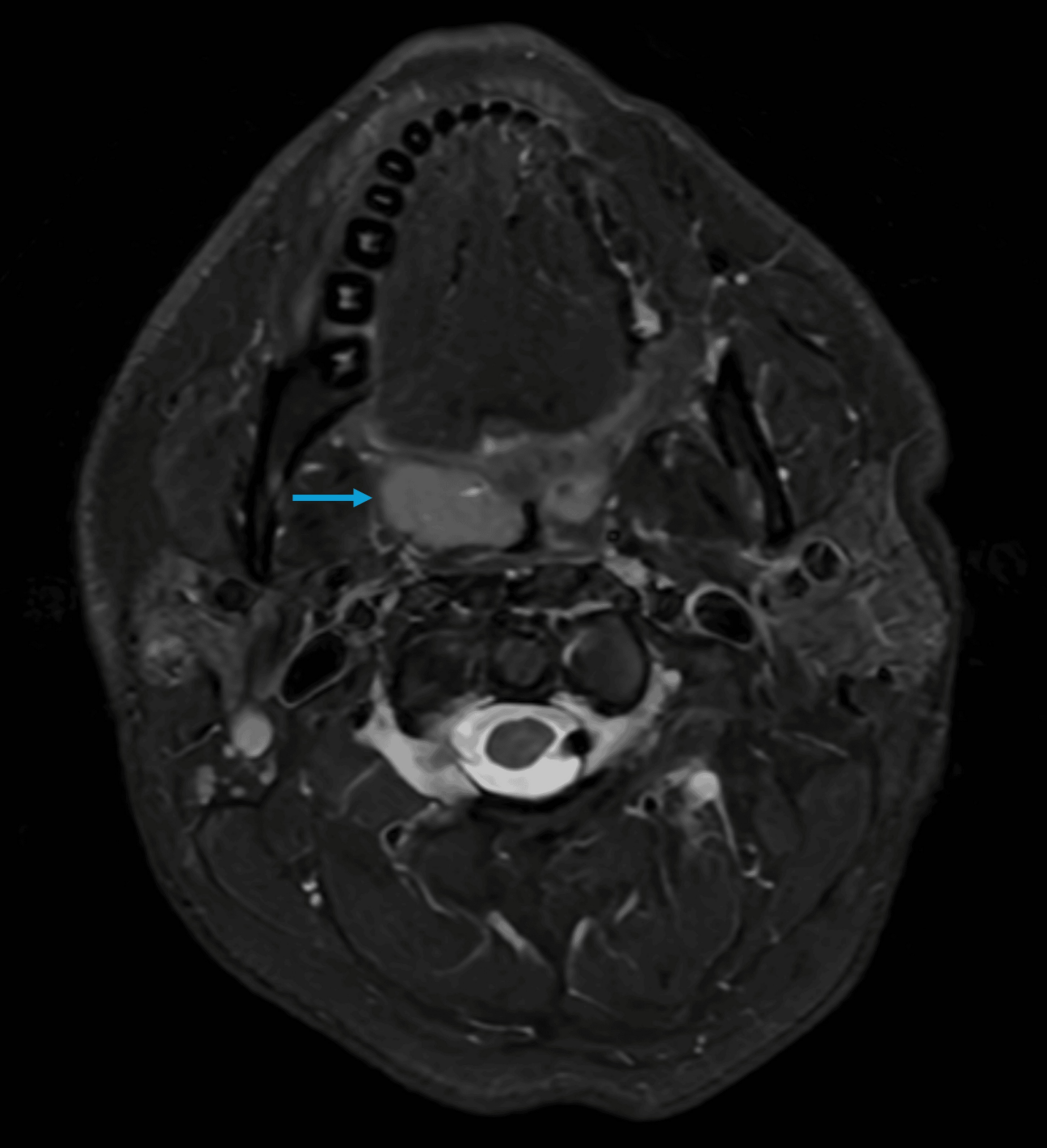
Asymmetric tonsillar appearance on contrast-enhanced MRI Contrast-enhanced axial MR image of the neck demonstrating asymmetric tonsillar enlargement, with the blue arrow indicating the enlarged right tonsil and no evidence of local invasion. MRI: magnetic resonance imaging

On examination, the patient had a Brodsky grade 3 right tonsil, which was firm on palpation, and a grade 1 left tonsil. The tonsillar enlargement was uniform and non-ulcerative. There was no palpable cervical lymphadenopathy, and flexible nasendoscopy demonstrated no other abnormalities. Neck ultrasound demonstrated no pathological lymphadenopathy. An incidental thyroglossal duct cyst was also identified. Magnetic resonance imaging (MRI) demonstrated asymmetrical tonsillar enlargement without evidence of local invasion or metastatic disease, shown in Figure 1. Positron emission tomography (PET) imaging confirmed increased bilateral tonsillar uptake, more marked on the right, as well as an incidental focus in the sigmoid colon. The patient was consented for bilateral tonsillectomy under general anaesthesia, while gastroenterology arranged repeat colonoscopy with biopsies.

Bilateral tonsillectomy was performed with bipolar dissection. Intraoperative findings were in keeping with the clinical assessment. Both tonsillar specimens were sent for histopathological analysis via the two-week-wait cancer pathway.

**Figure 2:**
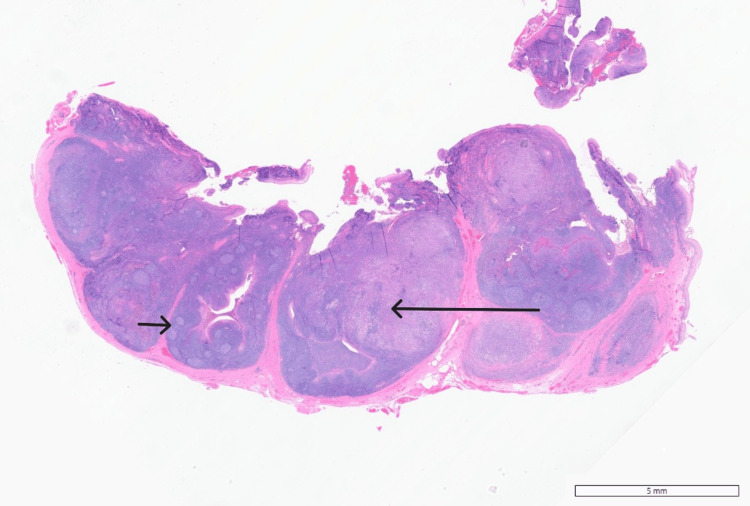
H&E stain (×0.5 magnification) This is a low-power image (×0.5 magnification) of the tonsillar tissue. It contains areas of normal tonsillar parenchyma (short arrow) demonstrating reactive changes (follicular hyperplasia) adjacent to ill-defined pale areas containing lesional cells (long arrow). H&E: haematoxylin and eosin

**Figure 3:**
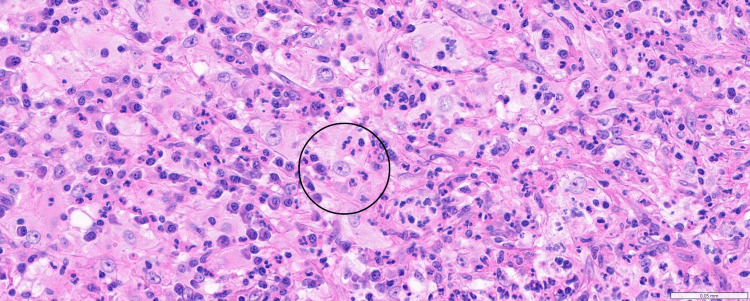
H&E stain (×40 magnification) This is a high-power image (×40 magnification) showing histiocytes and admixed inflammatory cells including lymphocytes, plasma cells, and neutrophils. The histiocytes have large, round to oval hypochromatic nuclei, prominent nucleoli, and abundant pale eosinophilic cytoplasm. Some of these contain engulfed and intact inflammatory cells (circle). This is a phenomenon known as emperipolesis which is characteristic of Rosai-Dorfman disease. H&E: haematoxylin and eosin

The specimens were reviewed by the Haemato-Oncology Diagnostic Service at a tertiary centre. Macroscopically, the right tonsil measured 3.5×2.2×1 cm, and the left tonsil measured 3×1×1 cm. Microscopic examination demonstrated extensive infiltration of the T-cell areas by large histiocytic cells with prominent emperipolesis (Figures 2-3). Immunohistochemistry was positive for S100 and CD68 and negative for CD1a, confirming the diagnosis of RDD.

Following the diagnosis, the patient was reviewed in the ENT clinic for further discussion and multidisciplinary planning. No postoperative complications were reported. He subsequently underwent successful removal of the submandibular duct stone. A referral to haematology was made, and he has since been followed up in the histiocytosis clinic. During follow-up, lung nodules were identified on computed tomography (CT) and monitored by respiratory medicine. In addition, multiple skin lesions excised by dermatology and urology were confirmed to represent cutaneous manifestations of RDD. He was also re-reviewed by gastroenterology, and repeat colonic biopsies were consistent with UC and negative for RDD. Notably, the patient's lymphadenopathy and cutaneous lesions resolved during corticosteroid therapy administered for a flare of UC, supporting the recognised steroid responsiveness of RDD.

## Discussion

Unilateral tonsillar hypertrophy is a common reason for referral to the ENT outpatient clinic and often raises concern for possible malignancy. RDD is a rare but important differential diagnosis in such cases. To our knowledge, three previous case reports have described tonsillar involvement in association with other manifestations, including cervical lymphadenopathy, nasal polyps, and tonsillar hypertrophy [3-5]. This case appears to be the first in the literature to suggest that RDD may present as isolated asymmetrical tonsillar enlargement.

RDD most commonly presents with massive cervical lymphadenopathy [6], whereas extranodal disease is less common. Reported extranodal sites include the respiratory tract, skin, nasal cavity, orbit, and bone [7]. RDD may present to the ENT clinic with a wide range of symptoms and anatomical manifestations. Head and neck radiological case series have demonstrated involvement of multiple sites, including the meninges, pituitary gland, lacrimal glands, paranasal sinuses, salivary glands, skin, tonsils, vertebral bodies, and thymus [8]. It has also been reported as a lytic lesion of the temporal bone [9]. Another case report published described a patient with an intracranial tumour, bony lesions, and bilateral cervical lymphadenopathy secondary to RDD [10], while another report documented laryngeal involvement [11]. However, the full spectrum of ENT manifestations of RDD remains poorly understood.

Although RDD generally carries a favourable prognosis, it may follow a relapsing course and has been associated with oncogenic mutations. Management is often conservative; however, long-term follow-up with haematology within a multidisciplinary setting is required.

## Conclusions

Tonsillar asymmetry has a broad differential diagnosis, and RDD represents a rare but important cause. Although it is generally associated with a favourable prognosis, RDD requires multidisciplinary management and close haematology follow-up. Greater awareness of this condition may improve clinician recognition and help guide subsequent multidisciplinary management.
